# Nacre-mimetic cerium-doped nano-hydroxyapatite/chitosan layered composite scaffolds regulate bone regeneration via OPG/RANKL signaling pathway

**DOI:** 10.1186/s12951-023-01988-y

**Published:** 2023-08-08

**Authors:** Xiao-Liang Liu, Chuan-Jian Zhang, Jing-Jing Shi, Qin-Fei Ke, Yu-Wei Ge, Zhen-An Zhu, Ya-Ping Guo

**Affiliations:** 1 Shanghai Key Laboratory of Orthopedic Implants, Department of Orthopedic Surgery, Shanghai Ninth People’s Hospital, Shanghai Jiao Tong University School of Medicine, Shanghai, 200011 China; 2 The Education Ministry Key Lab of Resource Chemistry, Shanghai Key Laboratory of Rare Earth Functional Materials, Shanghai Normal University, Shanghai, 200234 China; 3 Department of Orthopedic Surgery, Shanghai Jiao Tong University Affiliated Shanghai Sixth People’s Hospital, 600 Yishan Road, Shanghai, 200233 China

**Keywords:** Layered, Nacre-mimetic, Bone regeneration, Cerium, Hydroxyapatite nanosheets

## Abstract

Autogenous bone grafting has long been considered the gold standard for treating critical bone defects. However, its use is plagued by numerous drawbacks, such as limited supply, donor site morbidity, and restricted use for giant-sized defects. For this reason, there is an increasing need for effective bone substitutes to treat these defects. Mollusk nacre is a natural structure with outstanding mechanical property due to its notable “brick-and-mortar” architecture. Inspired by the nacre architecture, our team designed and fabricated a nacre-mimetic cerium-doped layered nano-hydroxyapatite/chitosan layered composite scaffold (CeHA/CS). Hydroxyapatite can provide a certain strength to the material like a brick. And as a polymer material, chitosan can slow down the force when the material is impacted, like an adhesive. As seen in natural nacre, the combination of these inorganic and organic components results in remarkable tensile strength and fracture toughness. Cerium ions have been demonstrated exceptional anti-osteoclastogenesis capabilities. Our scaffold featured a distinct layered HA/CS composite structure with intervals ranging from 50 to 200 μm, which provided a conducive environment for human bone marrow mesenchymal stem cell (hBMSC) adhesion and proliferation, allowing for in situ growth of newly formed bone tissue. In vitro, Western-blot and qPCR analyses showed that the CeHA/CS layered composite scaffolds significantly promoted the osteogenic process by upregulating the expressions of osteogenic-related genes such as RUNX2, OCN, and COL1, while inhibiting osteoclast differentiation, as indicated by reduced TRAP-positive osteoclasts and decreased bone resorption. In vivo, calvarial defects in rats demonstrated that the layered CeHA/CS scaffolds significantly accelerated bone regeneration at the defect site, and immunofluorescence indicated a lowered RANKL/OPG ratio. Overall, our results demonstrate that CeHA/CS scaffolds offer a promising platform for bone regeneration in critical defect management, as they promote osteogenesis and inhibit osteoclast activation.

## Introduction

A bone defect is a common feature occurring in fractures and diseases that originated from infection, tumor, trauma and osteotomies which decreases bone mechanical strength and impairs bone biological function [1–3]. The natural regeneration mechanism of the body enables the healing of bone defects without medical intervention, especially in young patients [2, 4]. However, for patients with larger defects or attenuated bone self-regenerative function, surgical intervention is required [3, 5, 6]. Critical bone defects not only bring motor disability, but also cause economic impacts and wear long-term outcome for patients [7–9]. Although, there has been several clinical managements for bone defects, the ultimate treatment was still controversial [7, 10, 11]. The gold standard in current bone defect management is autogenous bone grafting. However, with drawbacks of supply and morbidity from bone harvest, limitations on giant-sized defects, complications in donor sites, etc., there has raised a need for other bone substitutes [7, 12]. One of the main concerns regarding repairing bone defects is to maintain mechanical strength to avoid secondary injury, which requires the bone architecture to be stable enough when bearing mechanical force [13, 14]. Besides, the bone needs a method to promote new bone formation at the defect site by promoting the proliferation of osteogenic tissues, materials involved in the treatment should be associated with bone formation process [15, 16].

In recent years, various bone scaffolds such as bioglass (BG), hydroxyapatite (HA), β-tricalcium phosphate (β-TCP) and chitosan (CS) have been proposed [2, 17–22]. With noticeable biocompatibility and osteoconductivity, those scaffolds have been widely used in attempts of building composite bioscaffolds [23, 24]. Natural bone is a combination of organic-inorganic materials in which HA mineralized on collagen fibrils and it exhibits remarkable mechanical strength as well as natural nacre architecture [25, 26]. The preparation of natural high-performance structural materials such as nacre is highly promising for next-generation composites [27–30]. The nacre-inspired design is based on the formation of an ordered structure of hard-boosted platelets and soft polymers (natural mother-of-pearl contains 95 vol% inorganic platelets) [31–34]. Natural nacre has excellent mechanical properties due to its special structure, which is attributed to platelet pullout, crack bridging and flexing mechanism [32–34]. Primarily, a bone repair material should be biocompatible with native tissues [35, 36]. CS is a natural cationic aminopolysaccharide and recognized with its biocompatibility in tissue engineering [37, 38]. However, single CS scaffold lacks the strength to bear weight force in critical bone defects [39]. Inspired by natural nacre layer, our team combined the prepared HA and CS in previous studies to create an HA/CS composite material that mimics nacre layer and verified its strength [40]. HA/CS scaffolds are recognized for their biodegradability and present excellent biocompatibility to biological tissues [41]. HA/CS scaffold can provide an appropriate environment for newly formed bone tissues, making it highly promising for bone tissue engineering [42–44].

Despite the nacre-mimetic HA/CS composite scaffold satisfies the strength requirement for bone tissue engineering. To promote bone regeneration, the materials involved should be promotive in bone formation. Although there are many chemicals which have been demonstrated playing significant roles in osteogenic process, like insulin-like growth factor-1 (IGF-1), transforming growth factor-β1 (TGF-β1), these chemicals are highly fragile when exposed outside the *vivo* environment and unstable when constructed into scaffold [45–47]. Alternatively, a small number of rare earth elements exist in the human body, such as lanthanum (La), cerium (Ce) and gadolinium (Gd), participating in stem cell differentiation and tissue regeneration [48]. Studies have shown that cerium deposition in the human bone is relatively high, indicating that bones may be the preferred precipitation sites for elemental cerium [49–51]. Cerium ions are associated with bone metabolism, inflammation toleration, antioxidation and antitumor [52–56]. Cerium ions have been studied for their anti-inflammatory effects in rheumatoid arthritis (RA) patients in which cerium particles acted as a reactive oxygen species (ROS) scavenger in the cellular environment to relieve the inflammatory level and reduce bone loss [54]. Besides, it’s reported that as the ROS level reduces, processes of macrophages releasing inflammatory factors are abated, which is an essential pathway to activate inflammatory processes and promote bone resorption [55]. Previous studies have demonstrated that bio-scaffolds associated with cerium nanoparticles were able to inhibit RANKL-induced osteoclast differentiation to prevent bone resorption [57]. In addition, researchers also revealed the promotive capacity of Cerium element in osteogenic process, such as J. M. Li et al. found that ceria nanoparticles accelerated bone formation and enhanced endochondral ossification–based bone regeneration [53], and B. Lu et al. revealed that cerium oxide nanoparticles promoted osteoblast differentiation and proliferation through the ERK pathway [52].

In this study, we constructed the nacre-mimetic cerium-doped layered hydroxyapatite/chitosan (CeHA/CS) layered composite scaffolds and validated its potential for regulating osteogenic process and osteoclast differentiation in vivo and in vitro. we investigated the potential of CeHA/CS layered composite scaffolds for bone repair and regeneration, tested the biocompatibility of the scaffolds for hBMSCs in vitro and revealed osteogenic promotions on osteoblast differentiation by qPCR and western blot. The inhibitions on RANKL-induced osteoclast differentiation were also studied in vitro. Animal models were used to characterize their defect-repairing ability in vivo. The CeHA/CS layered composite scaffolds exhibited extraordinary potentials for bone regeneration.

## Materials and methods

### Preparation of CeHA/CS layered scaffolds

CeHA/CS layered composite scaffolds were prepared as follows: First, 2 g CS (≥ 75%, Shanghai RichJoint Chemical Reagents Co., Ltd., Shanghai, China) was added to 50 ml 2% acetic acid (Shanghai RichJoint Chemical Reagents Co., Ltd., Shanghai, China) and stirred for 3 h until CS was completely dissolved. After that, the bubbles were removed, and the solution was heated for 30 min, and the sonicated solution was poured into a 24-well plate and frozen in a refrigerator at − 20 ^o^C. The frozen CS solution was placed in a freeze-dryer and dried for 72 h. The dried CS scaffolds were then immersed in a mixed solution containing 0.09 M CaCl_2_ (≥ 96%, Shanghai RichJoint Chemical Reagents Co., Ltd., Shanghai, China), 0.01 M Ce(NO_3_)_3_·6H_2_O (≥ 99%, Shanghai RichJoint Chemical Reagents Co., Ltd., Shanghai, China), and 0.09 M Na_2_CO_3_ (≥ 99.8%, Shanghai RichJoint Chemical Reagents Co., Ltd., Shanghai, China) to deposit calcium carbonate on the CS scaffold. To form HA by mineralization, the composite was finally immersed in 0.02 M phosphate buffer solution (PBS, Tianjin haoyang Biological Manufacture Co., Ltd, Tianjin, China) for 7 days to obtain CeHA/CS layered scaffolds, cerium is compounded into the scaffold in the form of ions.

### Characterization

The morphologies and layered structures of CS, HA/CS, CeHA/CS and were detected by scanning electron microscopy (SEM; JSM-6380LV, JEOL, Japan). X-ray diffractometer system (XRD, D/max-III C, Rigaku, Japan) was performed at an acceleration voltage of 40 kV in the range 5°–60° (2θ). Fourier transform infrared (FTIR) spectroscopic analyzes (PerkinElmer, USA) were performed to detect functional groups in the range 4000–550 cm^− 1^. The compressive strengths of the HA/CS and CeHA/CS scaffolds (r = 0.75 cm, h = 1.1 cm) were tested using a microcomputer-controlled electronic universal testing machine (WDW-0.5 C, Shanghai Hualong Microelectronics Co. Ltd., China) at a compression speed of 5 mm/min. Calculate the compressive strength of the material according to the formula:

σ$$=\frac{\text{F}}{\text{A}}$$ (1)

σ—compressive strength (MPa).

F—Material stress load (N).

A—Stressed area (mm^2^).

The pore size distribution of CeHA/CH layered composites was measured by an automated capillary flow porometer (CFP-1500AX, PMI, USA). Thermo gravimetric analysis of CeHA/CS scaffolds was determined over the temperature range of 20 to 800 ℃ with a heating rate of 10 ℃⋅min^− 1^ .

### Ion release testing of CeHA/CS layered scaffolds

0.4 g CeHA/CS layered scaffolds was added to 10 ml deionized water; then, an ion release experiment was performed in a constant temperature shaker at 37 °C. At the corresponding time, 4 ml supernatant was taken out to measure its ion concentration by electron coupled plasma mass spectrometer, and then 4 ml deionized water was added to continue the experiment.

### Cell viability and adhesion

Cells were cultured with Dulbecco’s modified eagle medium (DMEM) formulated with the addition of 10% fetal calf serum (FCS) and 1% penicillin/streptomycin at 37 ℃. The MC3T3-E1 cells (Shanghai Institutes for Biological Science, Chinese Academy of Science Shanghai, China) were seeded into a 96-well plate with a density of 1 × 10^4^ cells per well. Extraction solution was collected from medium which was used to soak HA/CS and CeHA/CS scaffolds after 24 h to conduct in vitro experiments. Cells in the control group were cultivated in regular media as blank control. The media of extraction solution of HA/CS and CeHA/CS were used to substitute DMEM after 24 h. Cell counting kit-8 (CCK-8, Dojindo, Kumamoto, Japan) was used to detect cell proliferation after cultivated for 1, 2 and 3 days. Human bone marrow mesenchymal stem cells (hBMSCs) (Shanghai Rochan Biotechnology CO. Shanghai, China) were seeded in a 24-well plate along with a scaffold for the observation of cell adhesion. Each well contained one scaffold with 1 × 10^4^ cells and after 12-hour cultivation, cell-adhered scaffolds were washed with PBS and soaked in 2.5% glutaraldehyde (Beijing Solarbio Science & Technology Co., Ltd, Beijing, China) for 20 min for fixation. Then, scaffolds were washed with PBS three times and progressively dehydrated using 75%, 85%, 95% and 100% ethanol. The morphologies of hBMSCs were characterized by SEM (Siri on 200, Fei, Hillsboro, USA).

### Osteogenic capacity in vitro

hBMSCs were seeded in a 24-well plate at a density of 1 × 10^4^ per well and cultivated for 7 days with hBMSCs osteogenesis differentiation medium (Cyagen Biosciences, Guangzhou, China), which was also prepared as the extraction solution of HA/CS and CeHA/CS. Then, hBMSCs were fixed with 4% paraformaldehyde (Biosharp Life Sciences, Anhui, China) and washed with PBS three times, followed with ALP staining (ALP kit Hongqiao, Shanghai, China) and optical microscopy. Quantitative determination of ALP activity was performed with alkaline phosphatase assay kit (Beyotime Biotechnology, Shanghai, China). 50 µl/well samples were mixed with same volume of chromogenic substrate (p-nitrophenyl phosphate, Beyotime Biotechnology, China) and then incubated at 37 °C for 10 min. Finally, quantitative analysis on ALP protein was carried out by an enzyme-labeled instrument at 405 nm. Total proteins were assayed by BCA Protein Assay Kit. The ALP activity was determined by normalizing to the total proteins. Also, alizarin red staining was conducted at day 20 with alizarin red staining kit (Hongqiao, Shanghai, China) and was quantified by an enzyme-labeled instrument at 550 nm after dissolved in cetylpyridinium chloride. 4 × 10^6^ hBMSCs were seeded in a 6-well plate per well with osteogenic differentiation media of extraction solution of HA/CS and CeHA/CS and cultivated for 7 days. A real-time quantitative polymerase chain reaction (RT-PCR, Applied Biosystems, Foster City, CA) was used to analyze the expression levels of osteogenic genes. The total RNA was collected with a RNeasy Mini kit (Qiagen: Valencia, CA, USA). The mRNA expressions of genes including collagen type I (COL-1), runt-related transcription factor 2 (RUNX2) and bone morphogenetic protein 2 (BMP-2) as well as a standard reference gene (GAPDH) were detected and analyzed by 2-^ΔΔCt^ method. The designed primers were as follows:

GAPDH forward 5’-CACCACCATGGAGAAGGCCG-3’.

And reverse 5’-ATGATGTTCTGGGCAGCCCC-3’.

RUNX2 forward 5’-GACTGTGGTTACCGTCATGGC-3’.

And reverse 5’-ACTTGGTTTTTCATAACAGCGGA-3’.

COL-1 forward 5’- GAGGGCCAAGACGAAGACATC − 3’.

And reverse 5’- CAGATCACGTCATCGCACAAC − 3’.

BMP2 forward 5’- GGAACGGACATTCGGTCCTT − 3’.

And reverse 5’- CACCATGGTCGACCTTTAGGA − 3’.

The osteoblast-associated proteins of osteocalcin (OCN), RUNX-2, COL1 and P-Smad1/5 were characterized by the western blot method. hBMSCs were cultivated in a media of DMEM and extraction solution of HA/CS and CeHA/CS for three days. Then, radioimmunoprecipitation assay (RIPA) lysis buffer solution (Biosharp Life Sciences, Anhui, China) was used to soak cells for 20 min, and the lysed samples were collected in 1.5-ml microcentrifuge tubes. Samples were centrifuged at 12,000 rpm for 10 min, and supernatants were collected. Bicinchoninic acid assay (BCA, Biosharp Life Sciences, Anhui, China) was used to detect the concentration of supernatants. Detected samples were loaded in SDS-PAGE gel for electrophoresis and transferred to polyvinylidene difluoride (PVDF) membranes. After blocking in 5% milk for 1 h, the membranes were incubated with primary antibodies at room temperature for 4 h and then washed by TBST (Beyotime Biotechnology, Shanghai, China) three times. These membranes were incubated with secondary antibodies (Cell Signaling Technology, Shanghai, China) for 1 h. Finally, blotting results were checked by the Odyssey infrared imaging system (LI-COR Biosciences, Lincoln, NE).

### Inhibition of osteoclast differentiation in vitro

Bone marrow macrophages (BMMs) were flushed out from the tibiae and femurs of 4–6-week-old c57BL/6 mice (16–18 g) bought from the Animal Centre Research Committee of the Shanghai Ninth People’s Hospital (Shanghai, China). Cells were collected and cultured in α-MEM (Gibco; Thermo Fisher Scientific, Inc., Waltham, MA, USA) with 30 ng/ml M-CSF (R&D Systems, Inc., Minneapolis, MN, USA), 10% FCS and 1% penicillin‑streptomycin (Hyclone Laboratories Inc., Logan, UT, USA) at 37 ˚C. Media were changed every 3 days. After 7 days, cells were dissociated and reseeded in the plates of each experimental group, and 30 ng/ml M-CSF and 50 ng/ml RANKL (R&D Systems, Inc., Minneapolis, MN, USA) were used as media to stimulate osteoclast differentiation. After 7 days, cells were fixed with 4% paraformaldehyde and washed three times by PBS. Osteoclastic differentiation was observed by TRAP staining (Sigma Aldrich, Merck KGaA). The radiographs of TRAP-positive areas were reserved using Image-Pro Plus 6.0 (Media Cybernetics, Inc., Rockville, MD, USA). For f-actin analysis, cells were fixed with 4% paraformaldehyde and permeabilized with 0.1% (v/v) Triton-100 (Sigma Aldrich, Merck KGaA) and then stained with rhodamine-conjugated phalloidin (Cytoskeleton, Inc., Denver, CO, USA) at 37 ℃ for 1 h. F-actin rings were characterized by LSM5 confocal microscope (magnification: 10x; Carl Zeiss AG, Oberkochen, Germany).

### Animal experiments

15 Sprague-Dawley (SD) female rats (200–250 g, 6/group) were bought from Shanghai Experiment Animal Research Centre, and all experimental procedures were approved by the Animal’s Hospital of Shanghai Jiao Tong University. After injected intraperitoneally with sodium pentobarbital for anesthesia, SD rats were shaved overhead, and 5-mm diameter holes were drilled on the skull to create a bilateral critical-size calvarial-defect model. the scaffolds of HA/CS and CeHA/CS were then filled into the drilled space before the scalps were sutured. All rats were sacrificed after 12 weeks, and the calvaria skull caps were dissected and soaked in 4% phosphate-buffered formalin solution for 7 days. Then, micro-CT was performed using a micro-CT system (mCT-80, Scanco Medical AG, Switzerland) on soaked samples with parameters of a voltage of 90 kV, current of 88 uA and voxel size of 28 μm. 3d reconstruction was made by the micro-CT images. Bone mineral density (BMD) and new bone volume/tissue volume (BV/TV) values were analyzed as well. 3 and 21 days before the rats were sacrificed, fluorescent-labeled alizarin red (30 mg/kg, Sigma Aldrich) and calcein (30 mg/kg, Sigma Aldrich) had been injected into each rat. Labeled rats’ skulls were cut into 150-nm-thick slices by a microtome (Leica, Hamburg, Germany) and analyzed by confocal laser scanning microscope (Leica, Heidelberg, Germany, Alizarin red: 543/580–670 nm, calcein: 488/500–550 nm). The PC-based analysis system was used to quantify the mineralization rate (MAR). For morphologic observation, cranium samples were impeded in paraffin and stained with Masson’s trichrome.

### Statistical analyses

All data were from at least three independent replicated experiments and values were presented as the mean ± standard deviation (SD). Statistically significant differences were determined by Student’s t-test using the SPSS 16.0 software (SPSS Inc., USA). (* P < 0.05 or ** P < 0.01).

## Results

### Morphology of CeHA/CS layered scaffolds

**Fig. 1:**
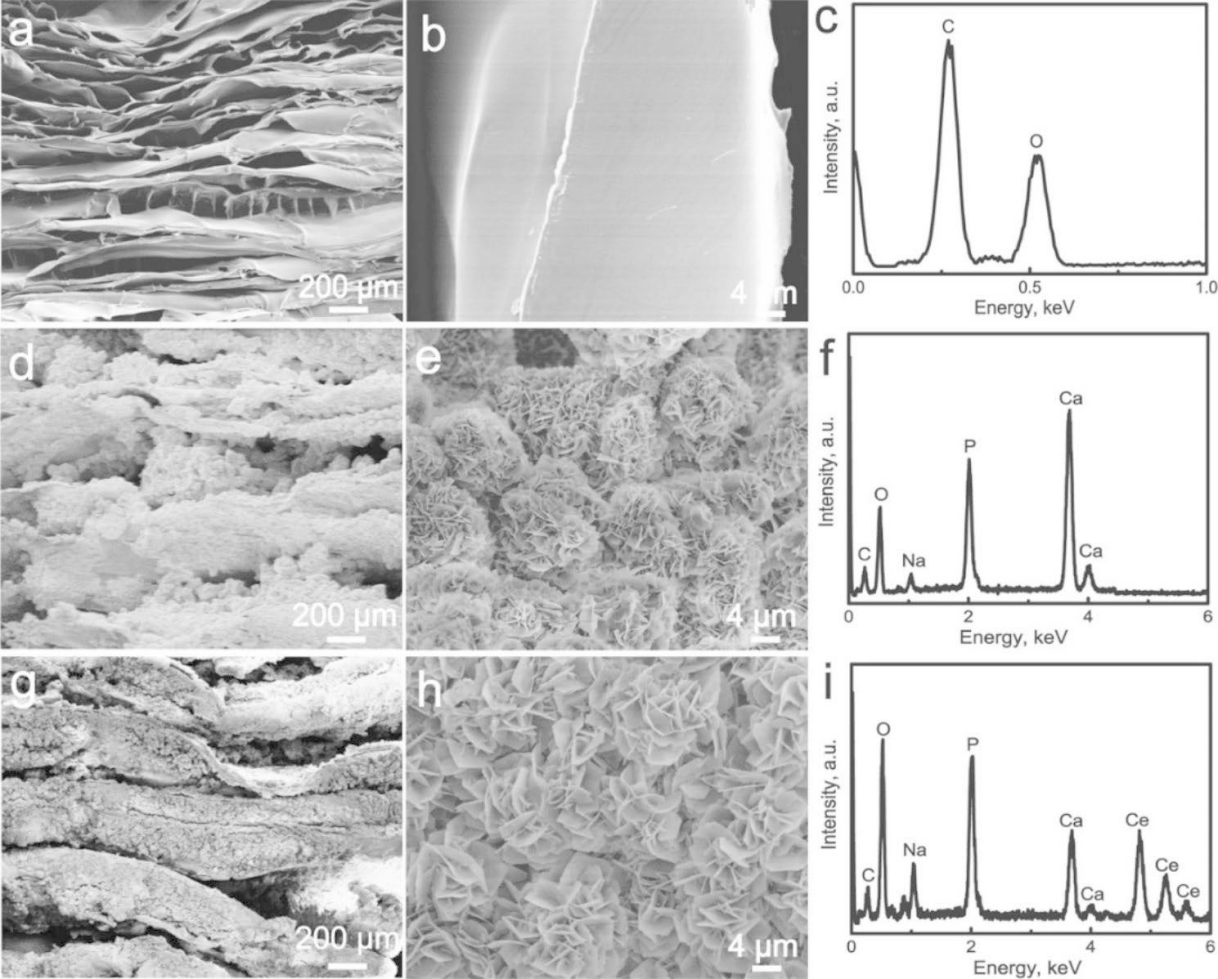
(**a**, **b**) SEM images of CS scaffold; (**d**, **e**) SEM images of HA/CS scaffold; (**g**, **h**) SEM images of CeHA/CS scaffold; (**c**, **f**, **i**) EDS spectrum

**Fig. 2:**
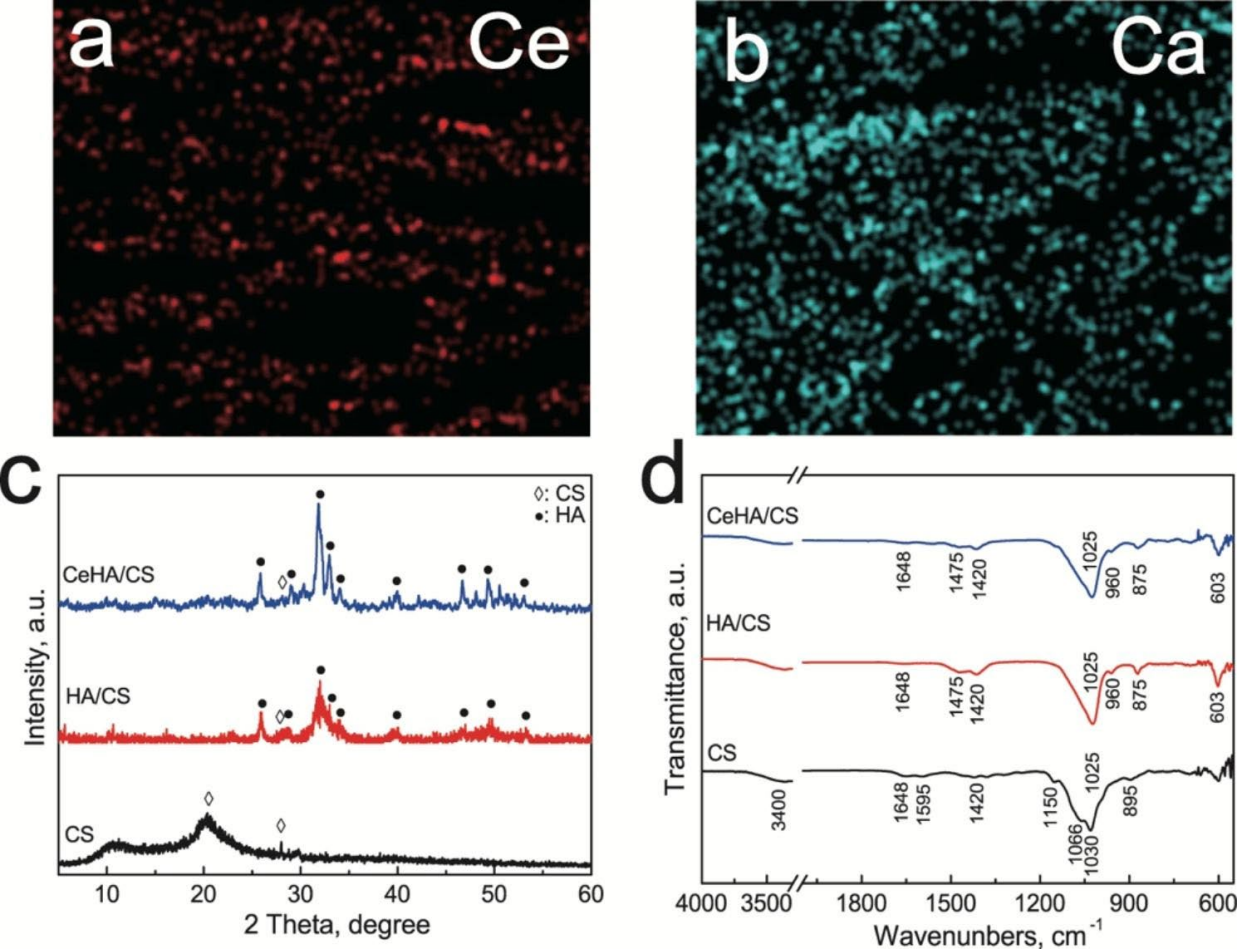
(**a**, **b**) Ce, Ca element distribution image respectively; (**c**) XRD and (**d**) FTIR spectra of CS, HA/CS, CeHA/CS.

By rapidly freezing CS at a higher temperature, the CS molecules will exhibit a certain orientation, and then placing the frozen CS solution in a freeze dryer to dry for 72 h to obtain a layered CS scaffold (Fig. 1a and b). The CS scaffold exhibits a layered structure (Fig. 1a). The high-resolution SEM image (Fig. 1b) indicates that the CS layer has a smooth surface. The EDS spectrum shows that CS mainly contains C and O elements. CS is a semi-crystalline organic matrix, which is confirmed by the peaks at 2θ = 20º and 28° in the XRD pattern (Fig. 2c). The FTIR spectrum of the CS layered scaffold also shows the characteristic vibration peaks of CS at 3400 cm^− 1^ caused by the OH- tensile vibration, and the band at 1648 cm^− 1^ corresponds to the amide I vibration, amino N–H deformation and oscillating vibration. The absorption peaks appear at 1595 and 895 cm^− 1^, while the absorption peaks at 1420 cm^− 1^ can be attributed to the vibration of C–N in the main group, the vibration at 1030 and 1066 cm^− 1^ to the tensile vibration of C–O, and the peak at 1150 cm^− 1^ to the tensile vibration of C–O–C group (Fig. 2d) [58].

In PBS, since there will be some hydrated calcium ions on the surface of CaCO_3_, these calcium ions will react with HPO_4_^2−^ to directly generate HA. The reaction equation is shown in (2). As the reaction proceeds, CaCO_3_ is converted to HA. Because a large amount of HPO_4_^2−^ is contained in PBS and the dissolution constant of HA is greater than CaCO_3_, CaCO_3_ will be converted to HA in situ in PBS. Therefore, we can immerse the CaCO_3_/CS layered composite in PBS so that CaCO_3_ attached to the layer can be converted into HA in situ. After doping with rare earth elements, the morphology of CeHA/CS layered scaffolds did not change, preserving a well-layered structure (Fig. 1g). High-resolution SEM images show that the HA converted by CaCO_3_ shows a flaky structure and 1–5 μm long (Fig. 1e h). The EDS chart shows that CeHA/CS layered scaffolds detected Ce elements compared to HA/CS. This indicates that rare earth elements have been successfully added to HA/CS scaffolds.

The transformed HA matched well with JCPDS card no. 09-0432 (Fig. 1i). By comparing the XRD (Fig. 2c) and FTIR (Fig. 2d) plots of HA/CS and CeHA/CS layered composites, the positions of the peaks and vibration peaks have not changed, indicating that the addition of rare earth elements will not change the scaffold crystal structure and composition. The FTIR plots of HA/CS and CeHA/CS layered composite show that the vibration of PO_4_^3−^ appears at 603 cm^− 1^ (v4), 960 cm^− 1^ (v1), 1025 cm^− 1^ (v3), 1648 cm^− 1^. And 1420 cm^− 1^ can be attributed to the amide I vibration and the vibration in the primary amide group of CS respectively [58, 59]. Interestingly, the vibrational fractions of CO_3_^2−^ were detected in the FTIR of HA/CS and CeHA/CS layered composite at 1475 cm^− 1^ and 875 cm^− 1^, respectively. This is because HA is converted from CaCO_3_, while CO_3_^2−^ may be partially retained in the crystal lattice of HA.

### Compression properties, ion release and TG analysis of CeHA/CS layered scaffolds

**Fig. 3:**
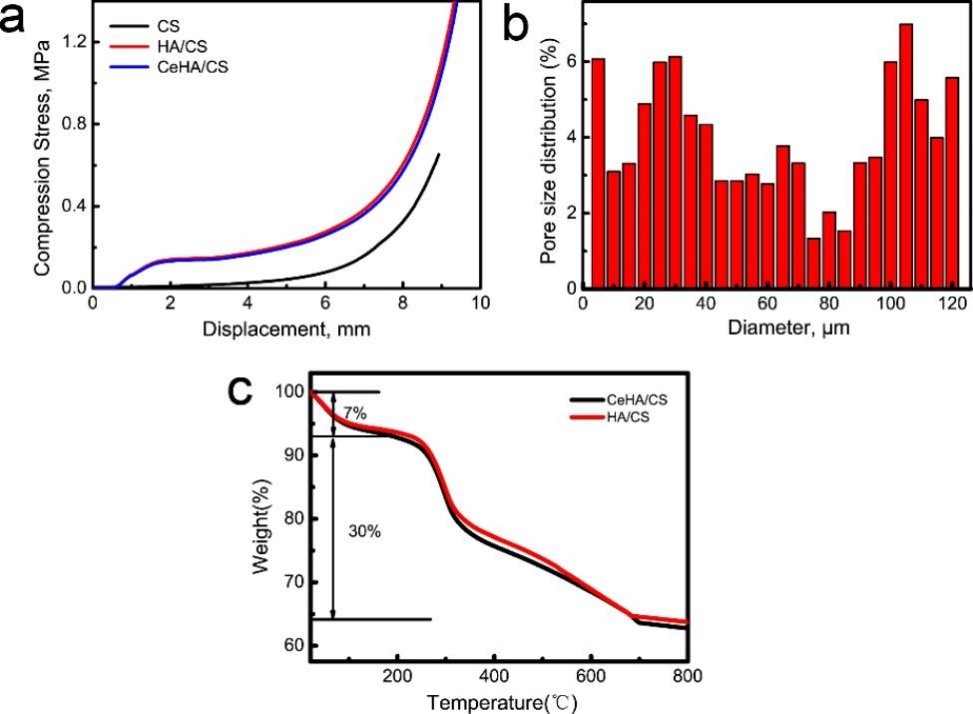
(**a**) Composite compression performance; (**b**) Pore size distributions of CeHA/CS; (**c**) TG analysis diagram of HA/CS and CeHA/CS.

Figure 3a shows that the force required to break the material structure is approximately 0.13 Mpa, deformation percentage is 18%. The main reason here is that HA can provide a certain support when the layered structure is destroyed. After that, the compressive force increases rapidly with the increase of deformation, because CS is a plastic material. When the deformation of CeHA/CS reaches about 80%, the compressive strength is 1.5 Mpa. HA/CS and CeHA/CS do not show much difference in compression performance, mainly because they are similar in structure. Table 1 shows the Young’s modulus of various materials, in which the Young’s modulus of CeHA/CS is 11.06 Mpa. This indicates that CeHA/CS can provide certain mechanical properties and is beneficial to bone tissue repair.

**Table 1:** Young’s modulus of various materials

Samples	Young’s modulus (MPa)	Reference
CeHA/CS	11.06	—
Chitosan biguanidine	3.2	[60]
Polyurethane/Chitosan scaffold	2.06	[61]
Gelatin	0.74	[62]
Gelatin-graft-poly (trimethylene carbonate)	2.43	[62]

The pore size distribution of CeHA/CS scaffold is shown in Fig. 3b. It shows that the pore size distribution of the CeHA/CS scaffold is mainly concentrated in 5–120 μm. Among them, the pore sizes of 5–50 mainly come from the voids between the lamellar HA, which is consistent with Fig. 1 h. The pore size of 50–120 μm mainly comes from the distance between the CS layers, which is consistent with Fig. 1 g. Meanwhile, according to Fig. 1 g, it can be seen that the maximum layer spacing is about 200 μm. Therefore, the distance between layers of HA/CS scaffolding is approximately 50 ~ 200 μm. Figure 3c is the TG analysis diagram of scaffold materials, and the quantitative analysis TG curve shows that the weight loss of materials mainly consists of two parts. Among them, the first part lost 7%, which is mainly due to the loss of water in the materials. The weight loss of the second part is 30%, which is mainly due to the decomposition of chitosan. The remaining weight is mainly hydroxyapatite, which is 63%. This is mainly because hydroxyapatite can exist stably at 800 ℃.

**Fig. 4:**
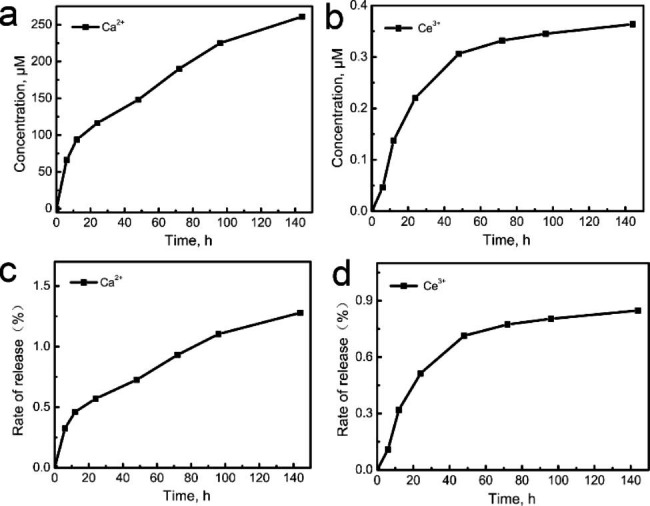
(**a**, **b**) Ion release curves of Ca^2+^ and Ce^3+^ for CeHA/CS respectively;(**c**, **d**) release rates of Ca^2+^ and Ce^3+^ for CeHA/CS respectively

The release characteristics of the Ca^2+^ and Ce^3+^ of CeHA/CS layered composite were studied by the ICP technique (Fig. 4). After soaking in ultra-pure water, Ca^2+^ and Ce^3+^ ions are released rapidly. With the increase of release time, the cumulative release of the Ca^2+^ and Ce^3+^ tends to be flat (Fig. 4a and b). After 144 h, the cumulative release of Ca^2+^ ions were 260.82 µM, and the cumulative release of Ce^3+^ ions was 0.36 µM. The relative ion release rates of Ca^2+^ and Ce^3+^ are 1.28% and 0.85% respectively (Fig. 4c and d).

### Cytotoxicity and adhesion of CeHA/CS scaffolds

**Fig. 5:**
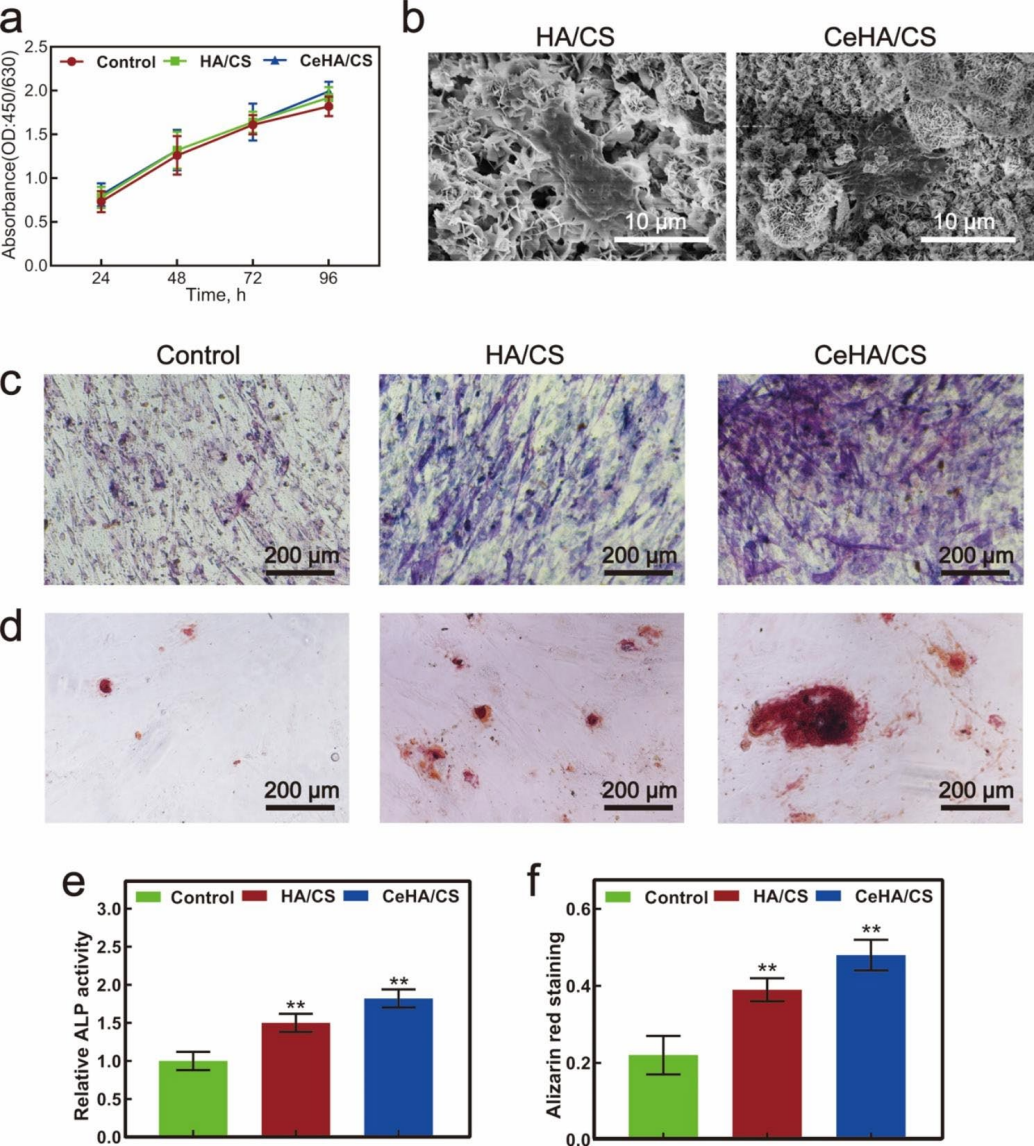
(**a**) CCK-8 analysis for control, HA/CS, and CeHA/CS scaffolds; (**b**) SEM images for HA/CS and CeHA/CS scaffolds adhered by hBMSCs for 12 h; (**c**) ALP staining and (**d**) alizarin red staining images of hBMSCs cultivated with control medium and extraction solution of HA/CS and CeHA/CS scaffolds for 7 and 20 days; Quantitative analysis of (**e**) relative ALP and (**f**) alizarin red activities of hBMSCs cultivated with control medium and extraction solution of HA/CS and CeHA/CS scaffolds. * P < 0.05, ** P < 0.01

The MC3T3-E1 cells were cultured in 96-well plates at a density of 1 × 10^4^ cells per well with an extraction solution of HA/CS and CeHA/CS for 1, 2 and 3 days and then tested for viability by CCK-8 kit. In the curve, cell numbers were continuously increased with time (Fig. 5a), and the number of cells nearly tripled after 96 h compared to that at 24 h. Intergroup curves have no distinct differences, indicating that these scaffolds did not attenuate MC3T3-E1 cell proliferation (Fig. 5a). The hBMSCs were seeded onto scaffolds and observed by SEM. The results showed hBMSCs adhered to the surface of scaffolds with protruded filopodia, and similar adhesions were found in each group; thus, scaffolds provided a suitable condition for cell-adhering capacity (Fig. 5b).

### Osteogenic promotion of CeHA/CS scaffolds in vitro

**Fig. 6:**
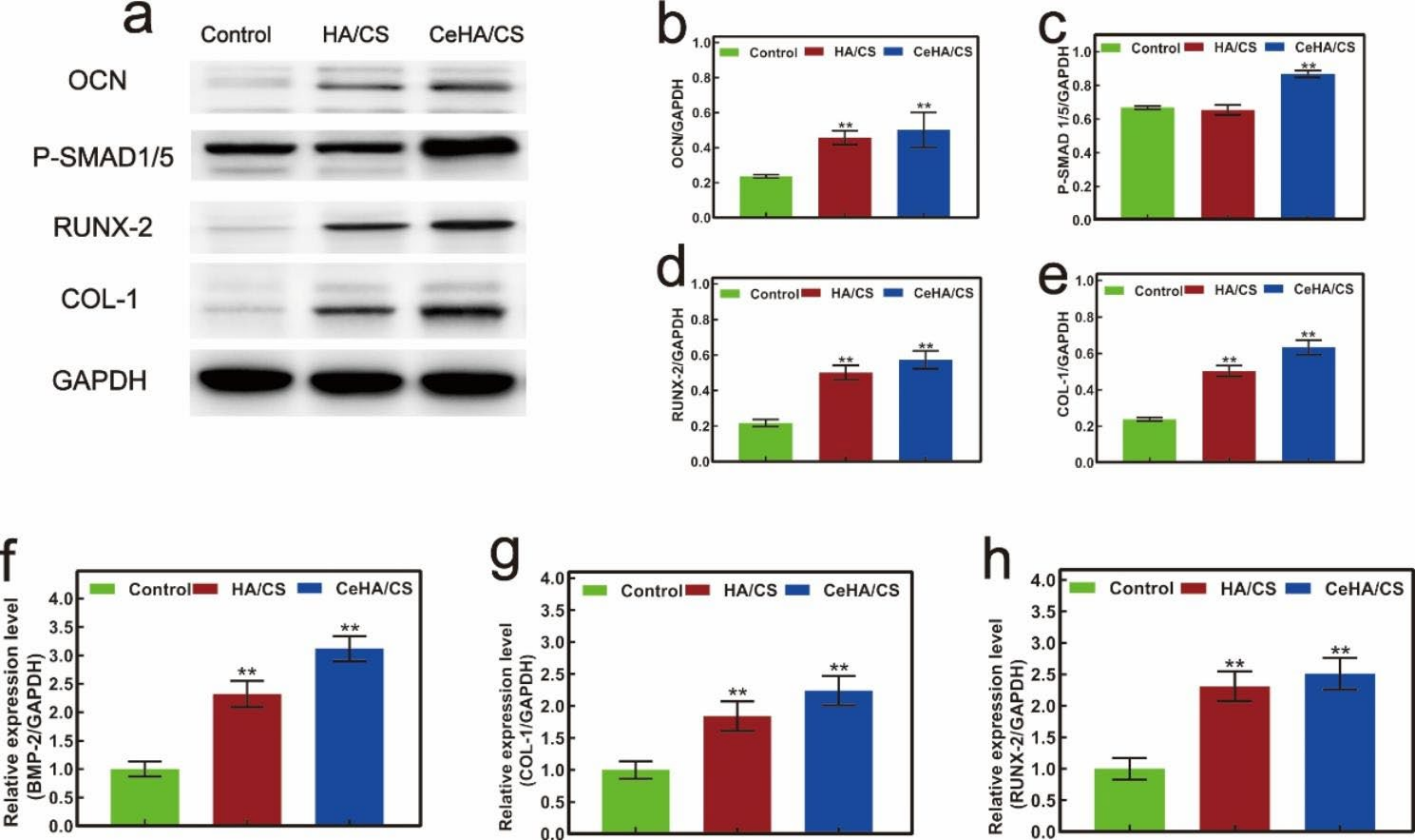
Western-blot analysis for expressions of osteogenic genes in hBMSCs cultured with control medium, extraction solution of HA/CS and CeHA/CS scaffolds: (**a**) Western-blot scanning images, quantitative western-blot analysis of (**b**)OCN, (**c**)P-SMAD 1/5, (**d**) RUNX-2, (**e**) COL-1 by ImageJ; qPCR analysis of relative expressions levels of osteogenesis-related genes: (**f**) BMP-2, (**g**) COL-1, (**h**) RUNX-2. * P < 0.05, ** P < 0.01

ALP staining was used to analyze the osteogenic effects of the scaffolds. In the control group, hBMSCs were cultivated with regular osteogenic differentiation media and meanwhile the cells in the HA/CS and CeHA/CS scaffolds groups were cultivated with the media of extraction solution of HA/CS and CeHA/CS scaffolds respectively. After cultivated with scaffolds and osteogenic differentiative solution for 7 days, the CeHA/CS scaffolds gained the highest ALP activity in comparison with HA/CS and control (Fig. 5c and e). Similar results were seen in alizarin red staining in which cells were cultivated with scaffolds for 20 days, the HA/CS and CeHA/CS scaffolds gained better alizarin activity than control, and the CeHA/CS scaffolds promoted the highest activity (Fig. 5d and f). Furthermore, the osteogenic capacity of scaffolds was investigated by RT-PCR, and the results revealed that HA/CS scaffolds doped with cerium promoted the expression of BMP-2, COL1 and RUNX2 compared with control group (Fig. 6f h). The osteogenic capacity was also validated by western blot analysis. CeHA/CS scaffolds presented the highest expression of OCN, COL1, RUNX2, P-Smad1/5 genes (Fig. 6a and e), which demonstrated osteogenesis-associated genes expressions were promoted.

### BMM differentiation inhibition of CeHA/CS scaffolds in vitro

**Fig. 7:**
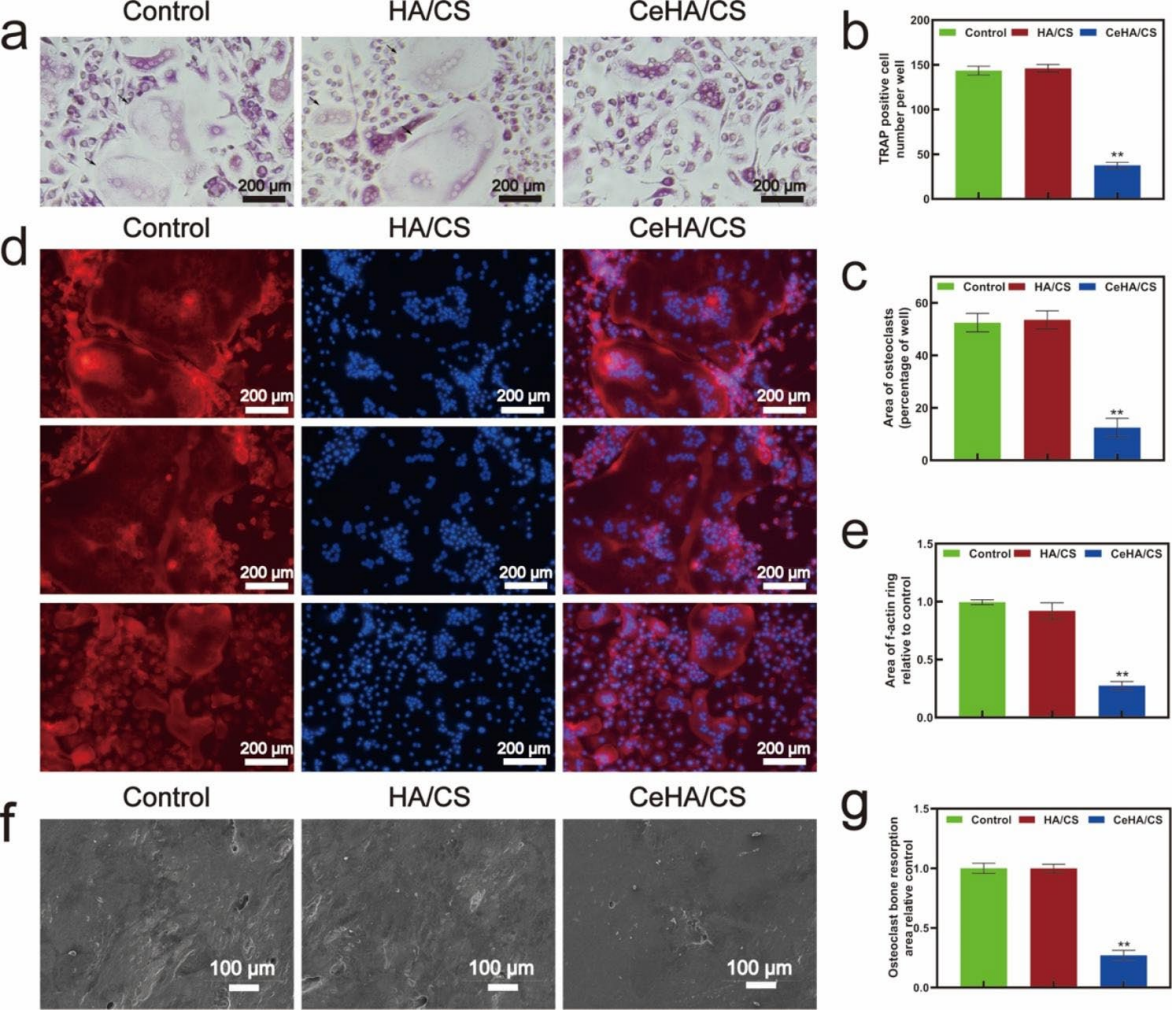
(**a**) TRAP staining in control, HA/CS and CeHA/CS scaffolds; (**b**) TRAP positive cell number per well and (**c**) osteoclasts percentage area measured by ImageJ; (**d**) F-actin immunofluorescence staining of cells in control, HA/CS and CeHA/CS scaffolds. (**e**) Measured area of f-actin ring compared to control by ImageJ; (**f**) SEM images of bone resorption area; (**g**) measured relative bone resorption area to control by ImageJ. * P < 0.05, ** P < 0.01

Cells in the control group and HA/CS scaffold obtained the highest TRAP activity but in the CeHA/CS scaffolds groups, it was relatively lower (Fig. 7a and c). Thus, the CeHA/CS scaffolds reduced osteoclast differentiation. However, HA/CS scaffolds didn’t affect the TRAP activity compared with control. CeHA/CS scaffolds also gained fewer f-actin rings compared with control and HA/CS scaffolds (Fig. 7d and f), which revealed the inhibitions on bone resorption activity. Generally, bone resorption process would be activated when pre-osteoclasts were differentiated into mature osteoclasts and induce bone loss. However, BMM cultivated with the CeHA/CS scaffolds were keep inactivated towards bone slices and the surfaces in CeHA/CS scaffolds group were obviously smooth (Fig. 7f). These results indicate that CeHA/CS scaffolds have inhibited osteoclast differentiation.

### In vivo rat calvarial defect models

**Fig. 8:**
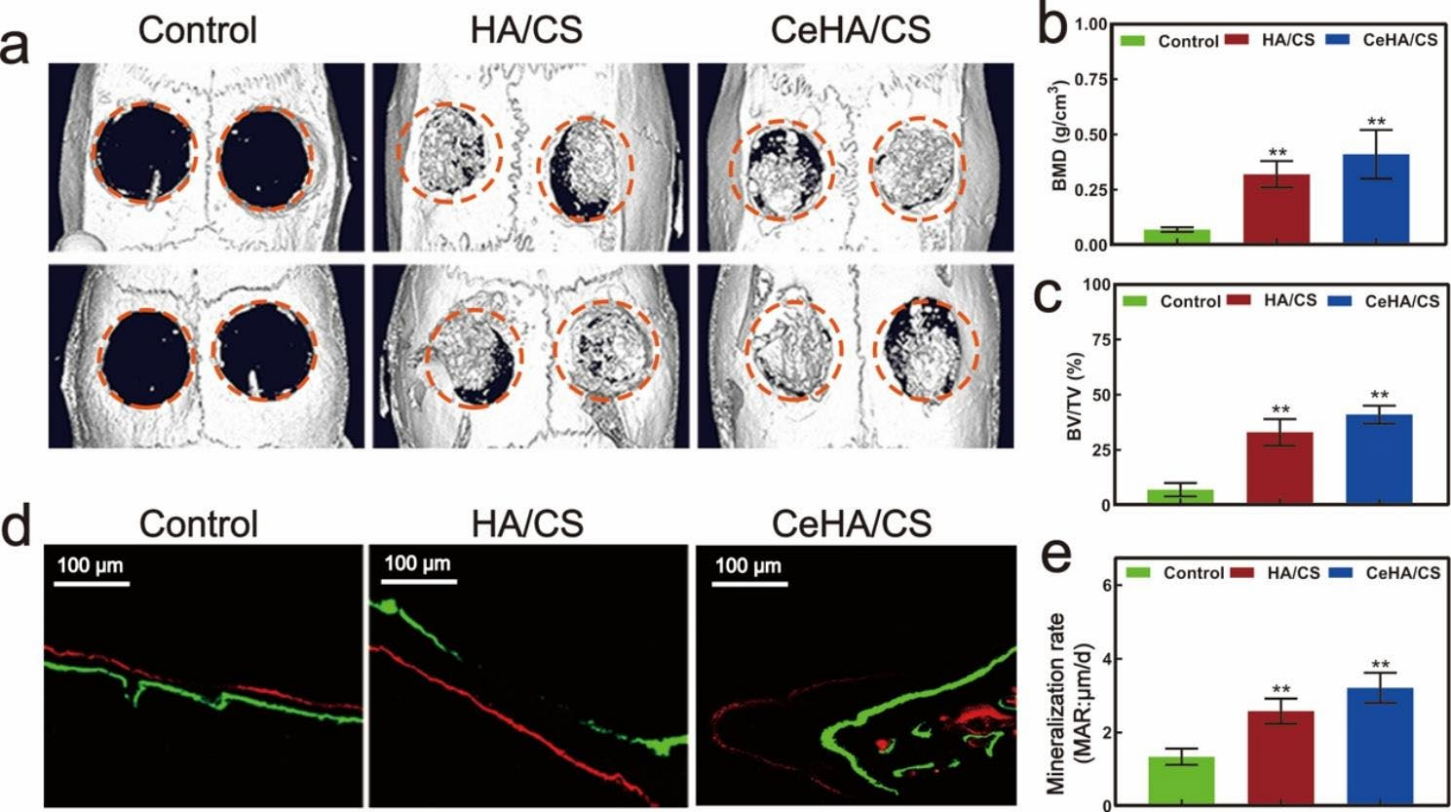
(**a**) Micro-CT images of rat calvarial defects in control, HA/CS and CeHA/CS scaffolds; (**b**) Bone mineral density; (**c**) Bone volume against tissue volume; (**d**) Fluorochromes marks of calcein (green) and alizarin red (red) injected 3 weeks and 3 days before euthanasia; (**e**) statistical analysis of mineralization rate. * P < 0.05, ** P < 0.01

The micro-CT images of calvarium revealed that rats with CeHA/CS scaffolds generated the most volume of newly formed bone compared with rats with control and HA/CS scaffolds (Fig. 8a). The same was observed for BMD: the BMDs in the CeHA/CS scaffolds were much higher than those in the other two groups (Fig. 8b). Also, the BV/TV values were higher in the CeHA/CS scaffolds, indicating CeHA/CS scaffolds remarkably contributed to the new bone formation in vivo (Fig. 8c). Fluorochromes marks indicated the growth in 18 days and rats with CeHA/CS scaffolds obtained the best growth rate (Fig. 8d and e).

**Fig. 9:**
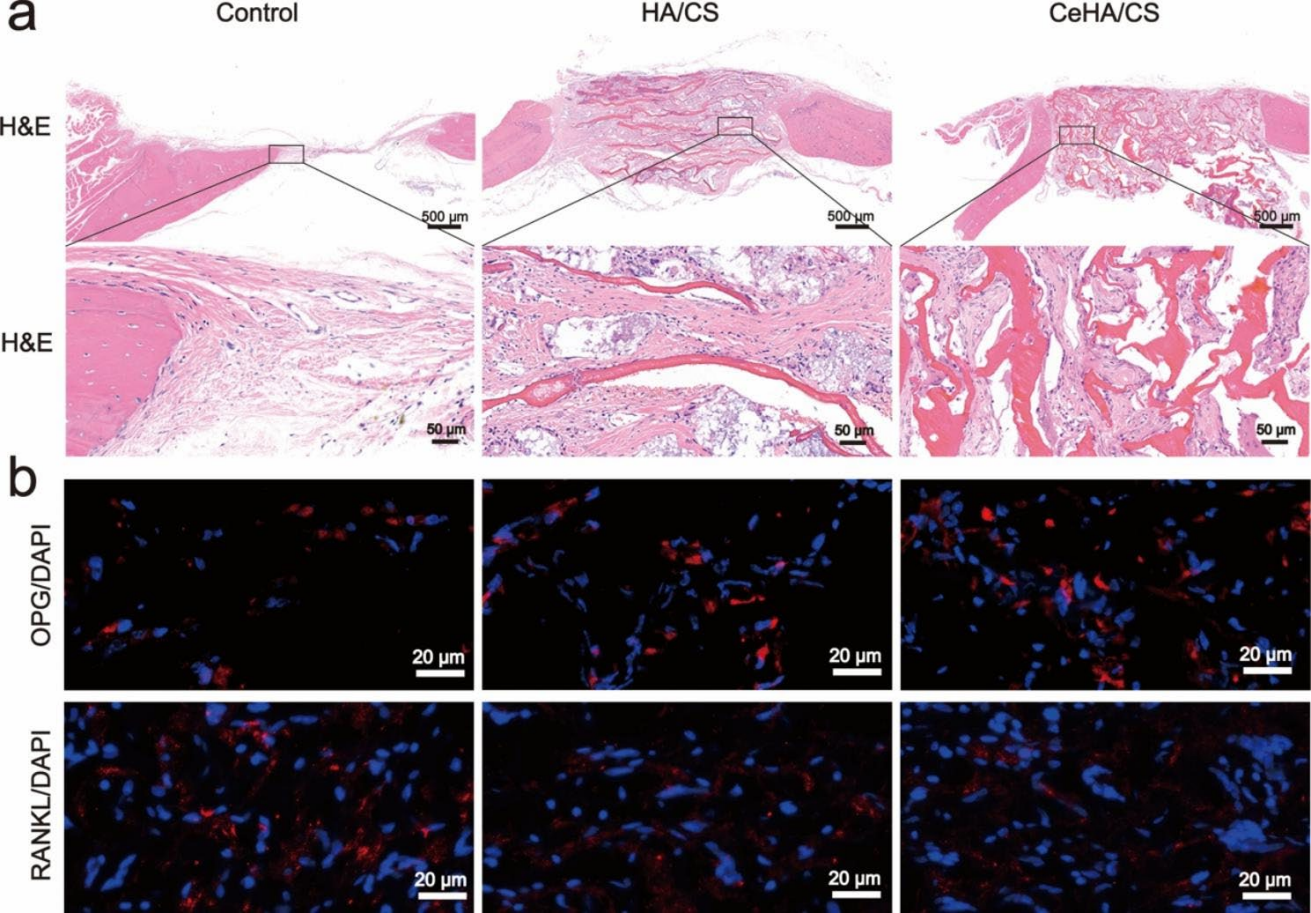
(**a**) H&E staining of the calvarial defect rats; (**b**) Immunofluorescence staining of DAPI (blue), OPG (red) and RANKL (red) from the calvarial defect rats

Histomorphology observations were made 3 months after calvarium defects were implanted with scaffolds by H&E staining. From the sagittal plane of the defects, it was observed that CeHA/CS scaffolds were accelerated in new bone formation inside the defects (Fig. 9a). Moreover, the influences of scaffolds on osteoclasts were analyzed by immunofluorescence labeling. The RANKL/OPG ratio was reduced in the CeHA/CS scaffolds in comparison with control and HA/CS scaffolds (Fig. 9b), which demonstrated the CeHA/CS scaffolds reconstruct bone tissue by promoting osteogenic activities and attenuated osteoclast differentiation.

## Discussion

Large bone defects have always been a major challenge in orthopedic procedures [1–3], and with damaged physical stability and impaired biofunction, defected bones are easy to cause fractures and difficult to perform surgical procedures [5, 6]. Various types of scaffolds have been studied for healing large bone defects, and scaffolds that promote new bone formation have been regarded as a prospective strategy [2, 3, 17–22].

Herein we created a scaffold with the strength of releasing osteogenesis-related elements to explore the possibilities of promoting bone regeneration. The CeHA/CS layered composite scaffolds were firstly prepared by dissolving CS into acetic acid, and after stirring and heating, the mixed solution was frozen and dried to form solid CS scaffolds. Then, dried CS scaffolds were immersed into the solutions of CaCl_2_, Ce(NO_3_)_3_·6H_2_O and Na_2_CO_3_, and calcium carbonate was generated on the CS scaffold doped with cerium. Fabricated scaffolds were then deposited with HA mineralization by immersing with PBS. Ultimately, the CeHA/CS layered composite scaffolds were successfully produced. SEM showed the scaffolds of CeHA/CS were arranged as layered structures (Fig. 1g), which shows that the addition of rare elements does not affect the original structure. The layered structures provided appropriate sites for osteoblast cells to contact and adhere (Fig. 5b). HA/CS scaffolds are biocompatible with living tissues, and no toxic effects were observed in this research (Fig. 5a). The layered structure provided an applicable bridge for cell migration and proliferation. CCK-8 results suggested that the CeHA/CS layered scaffolds have no toxic effects on MC3T3-E1 cells proliferation (Fig. 5a), and the SEM results indicate hBMSCs were stretched on the sheet-like surface and maintained steadily, adhering to the scaffolds (Fig. 5b). In the animal model, the sagittal plane of the defects displayed substantial penetrated connective tissues and newly formed bone structures (Fig. 9a), indicating the layered scaffolds provide excellent space for tissue growth in scaffolds. Penetrated tissues adhered to the scaffolds and proliferated (Fig. 9a), contributing to the growth of regenerated bones. Our work demonstrated that the regenerated bone tissues could prospectively adapt to our layered scaffolds.

Cerium is known as a rare element with significant positive functions, especially in the bone formation process [48]. It was reported that cerium can upregulate osteogenic gene expression and accelerate new bone formation [52, 53]. Herein, we generated the CeHA/CS scaffolds by incorporating cerium ions into the CeHA/CS scaffolds. In this way, cerium elements were within the CeHA/CS scaffolds, and meanwhile, the basic layered structure was preserved. We tested the CeHA/CS scaffolds’ osteogenic capacities and compared them with the previous scaffolds. It was shown that with cerium added to the HA/CS scaffolds, there was an obvious increase in ALP staining activity (Fig. 5c and e), indicating that CeHA/CS scaffolds significantly promoted the osteogenic activity. Similar results were observed in the alizarin red analysis. After 20 days of cultivation, the CeHA/CS scaffolds gained higher alizarin red activity (Fig. 5d and f), indicating improved calcium deposition. The results showed that the HA/CS and CeHA/CS scaffolds had excellent performances on ALP and alizarin red staining, while the CeHA/CS scaffolds performed better than the primary HA/CS scaffolds. Further, we studied the mechanism of osteogenic promotion in the presence of cerium by RT-PCR and western blot techniques. The expressions of osteogenic-associated genes of RUNX2, COL1, OCN and P-Smad1/5 significantly improved in the group cultivated with CeHA/CS scaffolds compared to the control and HA/CS scaffolds in western blots (Fig. 6a and e). The RT-PCR results revealed the same trend in the osteogenic mechanism as it upregulated the expressions of RUNX-2, BMP-2, COL1, indicating the osteogenic pathway was activated (Fig. 6f h). In the animal experiment, micro-CT showed the bone defects in the CeHA/CS group were better healed (Fig. 8a and c), and H&E staining indicated connective tissues and newly formed bones penetrated between layered structures (Fig. 9a).

In terms of bone resorption, cerium was known for acting as an ROS scavenger in the cellular environment [54, 56, 57, 63]. ROS in the cellular environment can lead to inflammation in tissues and may introduce osteoclast differentiation [55]. And cerium ions were proven to act as an anti-inflammation factor in macrophage polarization, which may inhibit pro-inflammation macrophage differentiation and eventually inhibited the osteoclast differentiation [54, 57]. In our study, CeHA/CS scaffolds attenuated the differentiation of BMMs into osteoclasts with downregulated TRAP activities (Fig. 7a) and successfully reduced bone resorption activity on the surface of bone slices (Fig. 7f). Also, in calvarial-defect rats, immunofluorescence histochemistry staining displayed a reduced RANKL/OPG ratio in the rats with CeHA/CS scaffolds implanted (Fig. 9b). The CeHA/CS scaffolds significantly accelerated new bone formation via osteogenic promotion and osteoclastic inhibition.

## Conclusion

The layered composite scaffolds were constructed by a freeze-dry strategy with Ce(NO_3_)_3_·6H_2_O dispersed in the compound solution. The CeHA/CS scaffolds provided an appropriate environment for cell proliferation and were characterized for their osteogenic functions owing to the upregulation of osteogenesis-related genes in hBMSCs. In addition, the CeHA/CS scaffolds inhibited osteoclast differentiation with fewer TRAP positive cells and attenuated bone resorption activities. Results in vivo revealed the promotive effects on new bone formation and mineralization in a rat calvarial defect model, and reduced RANKL/OPG ratio was observed as well. The CeHA/CS scaffolds exhibit remarkable potential for bone healing and regeneration.

## Data Availability

The data that support the findings of this study are available from the corresponding author upon reasonable request.
